# Assessment of Surface Sterilisation Approaches for the Removal of Pollen DNA from *Philaenus spumarius*

**DOI:** 10.3390/insects15100732

**Published:** 2024-09-24

**Authors:** Sam McGreig, Hollie Pufal, Chris Conyers, Eleanor P. Jones, Edward Haynes

**Affiliations:** 1 Fera Science Limited, York YO41 1LZ, UK; 2 School of Natural and Environmental Sciences, University of Newcastle, Newcastle NE1 7RU, UK

**Keywords:** surface sterilisation, insect gut content, plant DNA metabarcoding, pollen, meadow spittlebug, Aphrophoridae

## Abstract

**Simple Summary:**

To reliably identify the gut contents of plant-feeding insects, the removal of contaminant plant DNA from the insect surface is necessary. Previous approaches have used bleach and alcohol washes to achieve this. We perform a controlled baseline study on a herbivorous insect, the Meadow Spittlebug (*Philaenus spumarius*), to identify possible contamination that may persist after bleach washes. Despite the reported success of these methods, we find that contamination is still present, leading to unreliable results. We hypothesise that pollen is the main source of contamination, its robust nature making it difficult to remove. We conduct a further three experiments, investigating the effectiveness of more robust bleach washes, sterilised gut excision, and ultraviolet light as alternative sterilisation approaches. Overall, our findings indicate that we are unable to remove surface contamination while still detecting signals that may originate in the gut. In no experiment did we unequivocally detect plant DNA that originated in the *P. spumarius* gut.

**Abstract:**

Dietary analysis of herbivorous insects relies on successfully eliminating surface contamination. If this cannot be performed reliably, then it will not be possible to differentiate between plants that the insect is feeding on and plants the insect has been in contact with, either directly or via pollen. Methods in the literature often use bleach and alcohol washes to remove contamination. We perform a controlled metabarcoding baseline study on a herbivorous, xylem-feeding insect, the Meadow Spittlebug (*Philaenus spumarius*), using Oxford Nanopore Technologies (ONT) sequencing, and identify possible contamination that persists after washes. Despite the reported success of methods in the literature, we find that contamination is still present, leading to possible false-positive results. We hypothesise that pollen is the main source of contamination, its robust nature making it difficult to remove, and conduct a further three experiments with the goal of removing pollen from the surface of *Philaenus spumarius*. This study investigates the effectiveness of robust bleach/Tween/alcohol washes, sterile gut excision (including combined with Distel application), and ultraviolet light as alternative sterilisation approaches. Overall, our findings indicate that we are unable to remove surface contamination and still detect signals that may originate in the gut. In no experiment did we unequivocally detect plant DNA that originated in the *P. spumarius* gut.

## 1. Introduction

The diets of insects can offer insight into their feeding habits and movement patterns, which can be of use for invasive species and pest management [1], as well as for biodiversity [2] studies. Dietary analysis can be performed via DNA metabarcoding, which involves PCR amplification of target DNA from a taxonomic group of interest (e.g., plants for herbivorous insects), followed by high-throughput DNA sequencing of PCR products and comparison to an appropriate reference database. While this approach can allow the identification of DNA in gut contents, care must be taken that the DNA originates in the gut and not from the surface of the insect. This is less of an issue with predatory arthropods, where other animals are consumed [3], but plant DNA (in the form of pollen) is widespread in the environment (e.g., [4]). Therefore, where non-predatory insects are the focus of an insect diet study, it is a necessary step to mitigate the amount of surface-level contamination so that clear conclusions can be drawn about what the insect was feeding upon, as opposed to what external material it had come into contact with. There are a number of documented methods available in the literature that state they have successfully resolved the problem of external contamination of herbivorous insects with plant DNA. Cooper et al. [5] found that submerging insects in ethanol (70%) and bleach (0.5%), separated by rinses of deionised water, resulted in successful amplification of plant DNA that was assumed to be ingested material. Building upon Cooper’s method, Avanesyan and Lamp [6] submerged *Lycorma delicatula* nymphs in a 2% bleach solution and inferred that the recovered DNA was from ingested material. Similarly, Wallinger et al. [7] found that bathing wireworms in 1–1.5% bleach was enough to remove artificially introduced surface contamination. However, the effectiveness of these approaches has not always been rigorously assessed and may not reflect real-world surface contaminants. Linville and Wells [8] observed that soaking carnivorous maggots in bleach (20%) removed surface contamination whilst keeping the ingested material intact. This study was based on the use of Sanger sequencing, which may not pick up low-level contamination of the external spike into the gut contents. 

We report the effectiveness of surface-contaminant removal techniques on *Philaenus spumarius*, the Meadow Spittlebug, using high-throughput sequencing (HTS) as a means to identify plant material. We selected *P. spumarius* as our insect of interest, largely due to its ability to act as a vector for the transmission of the bacterium *Xylella fastidiosa* [9]. The identification of the feeding range of *P. spumarius* from ingested material would enable the targeted screening of plants, potentially resulting in the early detection of infected material. The detection of gut content DNA in *P. spumarius* is likely to be challenging, as this is a xylem-feeding insect that ingests xylem sap through a stylet inserted into the xylem [10], and low amounts of host DNA are likely to be present in the xylem.

In this study, our initial aim was to use HTS to identify the gut contents of *P. spumarius*. The results of this initial experiment led to further work to evaluate more rigorous surface-contaminant removal techniques, to evaluate gut dissection and Distel sterilisation, and to report on the potential of ultraviolet (UV) light sterilisation. The work presented here therefore comprises a series of complementary but separate experiments. 

Experiment 1 was contamination baselining. Metabarcoding was performed for gut content analysis of whole and parts of caged spittlebugs, using a published surface sterilisation method. We made use of a captive population of spittlebugs with a known, limited diet in order to confirm the functioning of the method. Our hypothesis was that we would be able to detect plant DNA in spittlebug samples originating from the food plants inside the cages. Experiment 2 was Tween and bleach sterilisation. This was an assessment of two further surface sterilisation techniques, using qPCR and metabarcoding of spittlebugs spiked with pollen. Our hypothesis was that the Tween and bleach sterilisation techniques would reduce detectable pollen to the level of the unspiked controls. Experiment 3 was gut dissection and Distel sterilisation to control surface contamination. This included the assessment of the excision and testing of spittlebug guts, as well as treatment with Distel, using metabarcoding of spittlebugs spiked with pollen. Our hypothesis was that the gut dissection and/or Distel treatment techniques would reduce detectable pollen to the level of the unspiked controls. Experiment 4 was UV sterilisation. This involved using qPCR to assess of the ability of ultraviolet light to destroy the DNA present in pollen. Our hypothesis was that UV treatment of pollen would render us unable to detect plant DNA using qPCR.

After detecting DNA in the first experiment that was inconsistent with the plants present in the controlled environment of the cages, we hypothesised that this was likely due to the presence of DNA from external sources in the form of pollen. We then designed the three subsequent experiments to try and identify a method to satisfactorily remove DNA from pollen on the exterior of the insects, allowing gut contents to be unambiguously identified.

## 2. Materials and Methods

### 2.1. Experiment 1: Contamination Baselining 

This initial experiment assumed contamination could be controlled using existing protocols and was designed to explore the diet of *P. spumarius*. Twenty *P. spumarius* were received, ten of which were reared on broad bean (*Vicia faba*), with the remaining ten reared on a mixture of lavender (*Lavandula augustifolia*), rosemary (*Salvia rosmarinus*), and broad bean. The *P. spumarius* were grown in closed mesh cages inside a glasshouse, with roof hatches opened to allow for cool air exchange from the outside. Three of the bean-fed and five of the mixed-fed *P. spumarius* were surface-sterilised using the technique outlined in [5]. Surface sterilisation involved submerging the individuals in 70% ethanol for 2 s, rinsing in deionised water for 2 s, submerging in 0.5% bleach for 1 minute, and rinsing twice in deionised water for 2 seconds each. Three of the bean-fed and five of the mixed-fed *P. spumarius* were not surface-sterilised. A further four non-surface-sterilised bean-fed *P. spumarius* had their heads and foreguts excised and their eyes removed [11] under sterile conditions in order to determine if the methodology was compatible with the EPPO protocol for detection of *X. fastidiosa*. This gave a total of 20 *P. spumarius* individuals for analysis. For this and subsequent experiments, DNA was extracted from these samples (whole insects or partially dissected as described) using a Qiagen Blood and Tissue kit. In the first step, samples were transferred into 1.5 mL microcentrifuge tubes containing 180 µL buffer ATL plus 20 µL 20 mg/mL Proteinase K and squashed with a sterile micropestle before incubation overnight at 56 °C and 350 rpm. Subsequent steps followed the manufacturer’s standard protocols. DNA was eluted in 100 µL buffer AE. All batches of extractions included a blank control.

A PCR was performed for the *rbcLa* marker gene to target plant DNA, using primers provided in Table 1. PCR reactions comprised 12.5 µL Q5^®^ Hot Start High-Fidelity 2× Master Mix (New England BioLabs Inc., Frankfurt am Main, Germany), 0.2 µM each of forward and reverse primer, and 5 µL DNA extract as template in a total volume of 25 µL. The extraction control and PCR-negative control (comprising 5 µL molecular biology-grade water) were also amplified alongside the samples to monitor for contamination. Cycling conditions were as follows: initial denaturation at 98 °C for 30 s, followed by 5 cycles of denaturation at 98 °C for 10 s, primer annealing at 49.5 °C for 40 s, extension at 72 °C for 1 min, followed by 35 cycles of 98 °C for 10 s, primer annealing at 54 °C for 40 s, extension at 72 °C for 1 min, with a final extension at 72 °C for 10 min and 12 °C hold. Following thermocycling, amplification success was measured by visualisation of amplicons on 1% agarose gels containing 0.1 µg/mL ethidium bromide (Sigma-Aldrich, Darmstadt, Germany). A total of 5 microlitres of the PCR reaction were added to 1 µL 6× Orange DNA Loading Dye (Fisher Scientific, Loughborough, UK) and electrophoresed on a 1% agarose gel in 1× TBE buffer for 1 h at 140 V. Amplicons were visualised on a UV transilluminator, and verification of correct amplicon size was carried out by comparison to a DNA size standard ladder (Quick Load DNA Marker Broad Range—New England BioLabs, Frankfurt am Main, Germany). 

PCR products were barcoded using the Oxford Nanopore Technologies (ONT) PCR barcoding (96) protocol for amplicons (SQK-LSK109 with EXP-PBC001 kits), and samples and DNA extraction control were sequenced on an R9.4.1 MinION flow cell. Basecalling was performed with Guppy version 5.0.11.

Bioinformatics analysis was performed as follows: Reads were trimmed with Cutadapt (version 3.2) [19], and then any reads that were too long (greater than 650 bases) or too short (fewer than 450 bases) for the expected amplicon size were removed. Subsequent filtering was applied to remove any reads that had a Phred quality score of less than 7 [20]. A custom *rbcLa* database was built from sequences obtained from Genbank. Briefly, curated rbcL sequences were obtained from [21]. These sequences were trimmed to the primers used in this study with Cutadapt [19], aligned with mafft (version 7.471) [22], and a hidden Markov model (HMM) was built using hmmer (version 3.3.2) [23]. The Rescript [24] software (version 2020.6) was used to download plant rbcL sequences from NCBI before extracting rbcL sequences with the HMM and filtering the sequences to remove those with too many degenerate bases, excessive homopolymers, and sequences that were too long/short. Finally, dereplication was applied to remove taxa with the same sequence and taxonomic annotation in the dataset. Individual reads were then subjected to a BLASTn (MegaBlast) [25] search against this database. The resulting reads were filtered so that only matches to sequences in the database that had a percentage identity of at least 80% and an alignment length of at least 80% were included. Of these hits, only the hits with a bitscore within 5% of the highest scoring hit were included in the final set of filtered reads. Finally, a lowest-common-ancestor approach was applied to the dataset, where if at least 75% of the assignments agreed, that taxonomic label was applied to the read. Otherwise, the next highest rank was considered, and the process was repeated until a label was assigned to the read. Where a lowest common ancestor could not be agreed upon, reads were assigned as ‘Unresolved’. For each sample, the taxon with the highest number of reads was selected, and then any taxon with a number of reads greater than or equal to 1% of this number was also selected. The reads associated with these taxa were extracted and used to build a higher-accuracy consensus sequence with Oxford Nanopore’s Medaka tool. Unassigned and unresolved reads and reads that were not used to generate consensus sequences were removed from subsequent analyses. Consensus sequences were manually inspected for assignments at the family, genus, and species level and collapsed or expanded to the genus level, where possible. Finally, samples with fewer than 50 total reads were removed.

### 2.2. Experiment 2: Tween and Bleach Sterilisation

**Figure 1:**
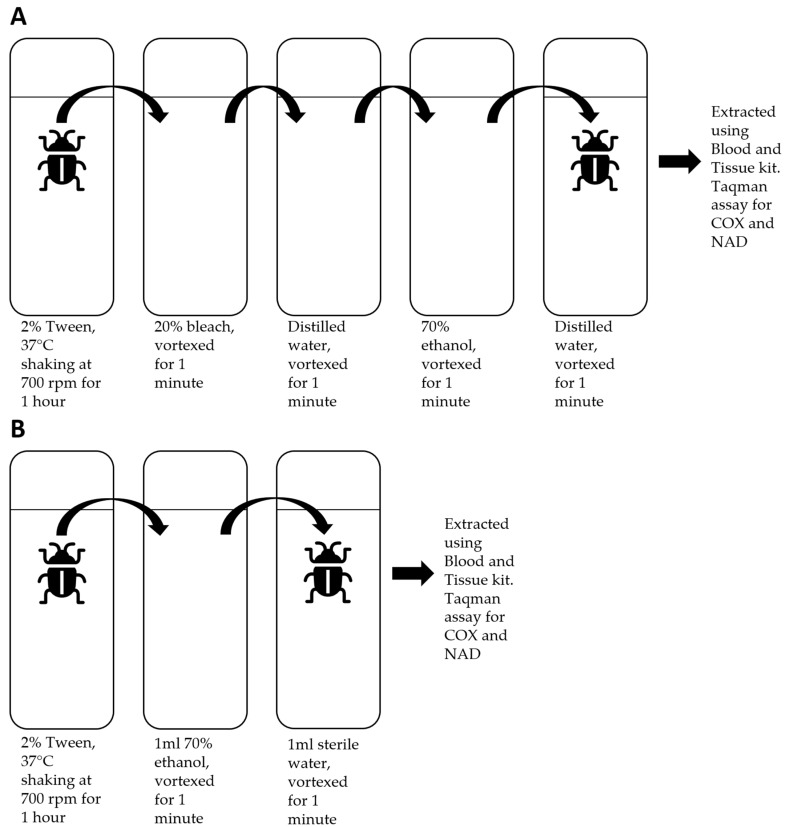
Illustrations of the treatments applied to each *P. spumarius* cohort. Cohort (**A**) consists of four *P. spumarius*, two coated in lily pollen and two uncoated. Cohort (**B**) consists of two *P. spumarius*, two coated in lily pollen and two uncoated.

After the initial experiment, it was determined we were unable to be confident that the results were from the gut contents rather than surface contamination (see Section 3.1). This experiment was designed to identify better decontamination methods, assessing these by dusting *P. spumarius* with pollen that could not have entered the spittlebug gut. Lily (*Lilium candidum*) was selected as the source of pollen for this experiment. Due to the season, it was highly unlikely that lily pollen would be present naturally. Eight *P. spumarius* that had been reared exclusively on broad bean were killed by freezing at −30 °C. Four of these individuals were coated in lily pollen by rolling in a Falcon tube containing the pollen, with the remainder left uncoated. Three of the coated and three of the uncoated *P. spumarius* were then submerged in a 2% Tween solution at 37 °C, which was shaken at 700 rpm for an hour. Tween^®^ 20 (Sigma-Aldrich, Darmstadt, Germany) is a detergent used to help remove pollen from the surface of *P. spumarius*. After an hour, two of the coated and two of the uncoated *P. spumarius* were vortexed in a 20% bleach solution for a minute, vortexed in distilled water for a minute, vortexed in 70% ethanol for a minute, and finally vortexed in distilled water for a further minute (Figure 1A). The remaining two Tween-treated *P. spumarius* were submerged in 1 mL of 70% ethanol and vortexed for a minute before being vortexed in distilled water for a further minute (Figure 1B). The remaining samples, one coated and one uncoated—which had not undergone any form of decontamination—were run as controls.

DNA was extracted from the whole spittlebugs as described above, and a TaqMan assay for COX and NAD was performed to monitor for the presence of plant DNA prior to sequencing, with NAD detecting lily DNA and COX detecting broad bean DNA. COX reactions comprised 12.5 µL Bio-Rad iTaq Universal Supermix 2.0 (Bio-Rad, Watford, UK), 0.3 µM each of forward and reverse primer, 0.1 µM probe, and 1 µL DNA extract as template in a total volume of 25 µL. Cycling conditions were as follows: initial denaturation at 95 °C for 2 min, followed by 40 cycles of denaturation at 95 °C for 15 s, and primer annealing/extension at 60 °C for 1 min. NAD reactions comprised 10 µL Bio-Rad iTaq One-Step reaction mix (Bio-Rad, Watford, UK), 0.05 µL Reverse Transcriptase mix, 0.3 µM each of forward and reverse primer, 0.1 µM probe, and 1 µL DNA extract as template in a total volume of 25 µL. Cycling conditions were as follows: 50 °C for 10 min, then 95 °C for 2 min, followed by 40 cycles of denaturation at 95 °C for 15 s and primer annealing/extension at 60 °C for 1 min. The extraction control and PCR-negative control (comprising 1 µL molecular biology-grade water) were also amplified alongside the samples in both assays to monitor for contamination. The sequences for the primers and probes for both assays are shown in Table 1. 

The four *P. spumarius* that were Tween-and-bleach-treated (two coated and two uncoated with pollen; Figure 1A) underwent a PCR amplification for the *rbcLa* marker gene using the primers described in Table 1 and methods described in Section 2.1 above. PCR products were barcoded using the ONT protocol PCR barcoding (96) of amplicons and sequenced on a Flongle (ONT, Oxford, UK). Bioinformatics analysis was performed using the same steps outlined in Section 2.1. 

### 2.3. Experiment 3: Gut Dissection and Distel Sterilisation to Control Surface Contamination

The results of experiment 2 (Section 2.2) suggested that it was extremely difficult to control surface contamination through surface decontamination steps. This third experiment was designed to explore the plant sequences arising from the surface of the insect (the exoskeleton) and to determine whether aseptic removal of the gut could exclude surface contamination and allow detection of ingested material. Again, the spittlebugs were dusted with a pollen control contaminant from a plant (tulip—*Tulipa gesneriana*) that could not have entered the spittlebug gut naturally as it was not present in the experimental chambers and was not in season at the time of the experiment. A total of 21 *P. spumarius* were reared on broad bean and underwent the treatments outlined in Table 2.

As the Tween and bleach solutions had been investigated as possible methods of surface sterilisation of the insects, a third option was assessed—the use of Distel (Tristel Solutions Ltd., Newmarket, UK), a high-level laboratory disinfectant with bactericidal, virucidal, fungicidal, and tuberculocidal activity that destroys both DNA and RNA. 

Three whole pollen-dusted and three whole non-pollen-dusted insects were each submerged in 1 mL 10% (*v*/*v*) Distel solution for 60 s with agitation at 700 rpm on a thermal mixer (ThermoScientific). They were transferred to 1 mL 70% ethanol and vortexed for 60 s, followed by transfer to 1 mL distilled water and vortexing for a further 60 s. DNA was extracted from all sample types (Table 2) using a DNA Blood and Tissue kit, as described in Section 2.1 above.

In contrast to experiments 1 and 2 above, PCR was performed using Illumina Nextera-tagged *rbcLa* (Table 1) in order to amplify plant DNA and to tag the samples for sequencing in a single PCR reaction. PCR reactions comprised 12.5 µL Q5^®^ Hot Start High-Fidelity 2× Master Mix (New England BioLabs Inc., Frankfurt am Main, Germany), 0.2 µM each of forward and reverse primer, and 5 µL DNA extract as template in a total volume of 25 µL. A PCR-negative control comprising 5 µL molecular biology-grade water was also amplified alongside the samples to monitor for contamination. Samples were amplified with the following thermocycling conditions on a BioRad T100 thermal cycler: initial denaturation at 98 °C for 1 min, followed by 40 cycles of denaturation at 98 °C for 30 s, primer annealing at 55 °C for 30 s, extension at 72 °C for 30 s, with a final extension at 72 °C for 10 min and 12 °C hold.

Following thermocycling, amplification success was measured by visualising amplicons on agarose gels as described in Section 2.1 above. 

Library preparation took place based on the Illumina protocol for 16S Metagenomic Sequencing Library Preparation (15044223 B Nov 2013, Illumina website). Firstly, the remaining 20 µL amplicon for each sample underwent a size-selection magnetic-bead clean-up to remove unincorporated PCR components and any small, non-specific products (e.g., primer dimers) using Agencourt AMPure XP magnetic beads (Beckman Coulter, Brea, CA, USA). Index PCR was performed using IDT for Illumina Nextera DNA Unique Dual Indexes. PCR reactions comprised 0.3 mM dNTPs, 5 µL indexes, 1 mM MgCl_2_, and 0.5 units Phusion^®^ High-Fidelity DNA Polymerase (New England BioLabs, Frankfurt am Main, Germany) in 1× HF buffer and 2.5 µL cleaned amplicon as template in a total volume of 25 µL. An index negative was included, which comprised 2.5 µL molecular biology-grade water. Samples were index-amplified with the following thermocycling conditions on a BioRad T100 thermal cycler: initial denaturation at 95 °C for 3 min, followed by 8 cycles of denaturation at 95 °C for 30 s, adapter annealing at 55 °C for 30 s, and extension at 72 °C for 30 s, followed by a final extension at 72 °C for 5 min and hold at 12 °C. Indexed samples (or ‘libraries’) then underwent a second magnetic-bead clean to remove unincorporated PCR components. The qualities of the libraries were then assessed by quantifying all libraries using a Quant-iT™ Picogreen™ dsDNA Assay Kit (Invitrogen, Waltham, MA, USA) and measuring library concentration on a Fluoroskan Ascent plate reader (Thermo Scientific, Vantaa, Finland). In addition, a selection of high- and low-quantifying libraries plus all controls (i.e., PCR-negative, extraction blanks, index PCR-negative) were run on an Agilent Technologies TapeStation 2200 using HS D1000 tapes, a size ladder, and sample buffer. 

Once the quality of the libraries had been assessed, the libraries were pooled in equimolar amounts to create a 20 nM library pool in a total volume of 250 µL. The pool was quantified using a Qubit™ dsDNA HS Assay (Invitrogen) and measured on a Qubit™ fluorometer to determine the actual concentration, and the average size of the pool was determined by running it on the TapeStation (Agilent, Stockport, UK). These were then used to dilute the library pool to 4 nM in preparation for running on the Illumina MiSeq (Illumina, Eindhoven, the Netherlands). Libraries were then sequenced on an Illumina MiSeq sequencer using the MiSeq Reagent Kit V3 (600 cycles). A total of 10 pmol sample pool and 10% PhiX were loaded onto the machine for sequencing. 

Samples were analysed using the Qiime2 software (version 2021.2) [26]. Samples had their primers removed with Cutadapt [19] and were subsequently denoised with Dada2 [27] to produce Amplicon Sequence Variants (ASVs). An additional round of chimera checking was then performed with Vsearch [28]. ASVs were taxonomically classified using a naïve-bayes classifier trained on *rbcLa* sequences obtained from the nuccore database using the Rescript software (version 2020.6) [24]. Samples that had fewer than 3000 ASVs assigned were removed from the analysis. Finally, a matrix was produced detailing the number of ASVs associated with each identified plant.

### 2.4. Experiment 4: UV Sterilisation

Lily pollen (*Lilium candidum*) was stored in a suspension of distilled water, which was subsequently vortexed to ensure a homogenous mixture. Six samples of 5 mL aliquots were then extracted from the mixture, and each aliquot was filtered using filter paper. The filter paper containing the pollen was placed in a weigh boat, pollen-side up, before being placed in the UV transilluminator (1200 J/cm^2^) for 0, 15, 30, 60, 120, and 240 min each. DNA was extracted from these samples using a Qiagen DNAeasy Blood and Tissue kit, following the manufacturer’s standard protocol (see Section 2.1 above), and an NAD TaqMan assay was run (see Section 2.2 above) to see if the pollen DNA was destroyed by the UV treatment.

## 3. Results

### 3.1. Experiment 1: Contamination Baselining

#### Sequencing Results

A total of 1,831,383 reads were generated, equating to just under 1.3 gigabases. The median read length of 597 was consistent with the expected *rbcLa* amplicon size (~598), and the average PHRED quality score of 13.4 equated to a 4.6% error rate. After primer trimming and PHRED quality score filtering, ~23% of the total reads were discarded, leaving 1,409,177 reads for downstream analysis. Two samples (one decapitated, non-sterilised, bean-fed; and one whole insect, sterilised, mixed-fed) failed to produce enough data and were excluded from analysis. The average number of reads per sample was 67,190, with a minimum read count of 2503 and a maximum read count of 145,283. The control samples produced 147 and 274 reads—numbers that were similar to the number of reads assigned to unused barcodes, suggesting that contamination was not a problem.

**Figure 2:**
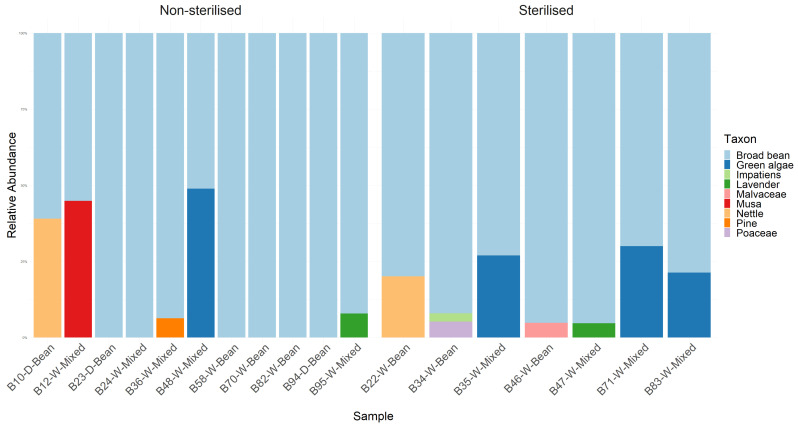
Samples from experiment 1, showing the relative abundance of DNA read counts per identified taxon per sample. The samples are split by treatment (sterilisation). The food source and dissection method are shown in the sample name (W = whole insects, D = decapitated). Two of the samples failed to produce sufficient sequence data and were excluded from the analysis.

Sequences identified as broad bean were detected in all samples that passed quality control checks, and lavender and rosemary were detected in two and zero samples from the ‘mixed diet’ cages, respectively, initially suggesting that broad bean was a preferred food source for *P. spumarius*. Additionally, a number of non-dietary plant sequences were detected in both the surface-sterilised and non-sterilised treatment groups (Figure 2). These reached relatively high proportions of DNA in some samples, with three of the nine samples reared on broad bean containing a >5% read count of ‘contaminating’ plant species and six of the nine samples reared on mixed plants containing > 5% read counts of ‘contaminating’ plant species. In the sterilised group, nettle (*Urtica*), mallow (Malvaceae), grass (Poaceae), and impatiens (*Impatiens*) were all detected. In the non-sterilised group, nettle, banana (Musa), and pine (*Pinus*) were detected. Green algae were also identified in the mixed-diet treatment group. Given the known identity of the plants in the *P. spumarius* cages, it was theorised that pollen from an open (meshed-covered) window could be the source of the external contamination. Of particular note is the detection of banana sequence—although not native to the UK, banana plants are grown in greenhouses at Fera, and a *Musa* species can be grown ornamentally outdoors in the UK.

Based on the results of this experiment, we determined that we had not sufficiently controlled surface contamination and could not distinguish between surface contamination and gut contents.

### 3.2. Experiment 2: Tween and Bleach Sterilisation

The purpose of this experiment was to test whether surface sterilisation with Tween or bleach was sufficient to remove surface contamination. All samples were tested with Taqman (Section 3.2.1), and the Tween/bleach samples were also analysed with metabarcoding (Section 3.2.2).

#### 3.2.1. Tween and Bleach Sterilisation TaqMan Results 

The negative NAD (CT value of 40) for the bleach-treated samples suggests that the pollen plant material was effectively removed from the samples. However, the COX values, which theoretically detect broad bean in the diet, are also high or negative, implying that a substantial amount of gut material was also removed (Table 3).

#### 3.2.2. Tween and Bleach Sequencing Results

A total of 27,146 reads were generated, equating to just over 6.2 megabases. The median read length of 190 was shorter than the expected *rbcLa* amplicon size (~598), and the average PHRED quality score of 12.1 equated to a 6.2% error rate. After primer trimming and PHRED quality score filtering, ~98.9% of the total reads were discarded, leaving 304 reads for downstream analysis. None of the remaining sequences were close matches to any reference taxa in the database, and manual inspection of a number of sequences revealed matches to ONT adapter sequences. There was therefore no sequenced plant DNA from the bleach-and-Tween-treated spittlebugs. The results confirm those of Section 3.2.1, where very little or no detectable DNA was observed.

### 3.3. Experiment 3: Gut Dissection and Distel Sterilisation to Control Surface Contamination

A total of 6,689,372 paired-end reads were generated, resulting in 3,732,800 ASVs (504 unique ASVs) after filtering from quality control steps. The extraction blanks and the PCR-negative controls were removed after quality control steps (with 11 and 0 reads, respectively). The average number of reads after filtering and quality control per sample was 188,332, with a minimum of 42,326 and a maximum of 287,844. Tulip was not detected in the whole and gut insect samples, where it was not spiked. It was, however, detected at low levels in the insect exoskeleton samples where it was not spiked, suggesting low-level contamination occurred during some of the lab processes, illustrating the difficulty of removing all sources of contamination in such sensitive analyses (Table 4). Where pollen was applied, it dominated (91–99%) the identified sequences (Supplementary Materials S1, E3—Matrix). This dominance was slightly lower in the Distel-treated samples, where tulip pollen accounted for between 40 and 55% of the identified DNA. 

Notably, the gut dissections revealed no presence of bean (the plants *P. spumarius* were reared on); instead, the presence of green algae was detected. This may not be surprising, given that *P. spumarius* are xylem feeders and so may ingest very little plant DNA [29]. The taxa identified in other sample types included bean, nettle, pine, conifer (Cupressaceae), and grass, a list of organisms overlapping with those identified in Section Sequencing Results. 

### 3.4. UV Sterilisation

Prolonged exposure to UV appeared to reduce the levels of lily pollen, as reported by the increasing CT values. Despite the small sample size, we observe a positive correlation between pollen degradation and the amount of time exposed to UV light—as time under UV increases, the levels of detectable pollen decrease. However, even after 240 min exposure, detectable pollen DNA remained (Table 5).

## 4. Discussion

Within our experiments, we initially attempted to determine the diet of a captive population of the spittlebug *P. spumarius* using DNA metabarcoding. Had this proved successful, the method would have been appropriate to roll out to wild-caught individuals to investigate the diet of *P. spumarius*. Indeed, the results for experiment 1 (and subsequently experiment 3) gave superficially plausible results, suggesting that, within the cages, the primary diet was broad bean, with occasional feeding on other plant species. However, both experiments were undertaken in rearing cages that contained either plants of broad bean only or plants of broad bean, lavender, and rosemary. The presence of plant DNA from species not found in the cages strongly suggested environmental contamination. These species were identified in two independent experiments (1 and 3) using different sequencing technologies—making these likely to be real observations as opposed to sequencing artifacts—and are highly likely to represent environmental contamination. We are confident the results are not laboratory-introduced contamination because the negative controls contained very low amounts of DNA and were removed after quality control steps due to low read counts. The contaminants identified have overlapping [30] pollen seasons, and we propose that surface contamination in the samples originates from pollen. Banana was identified in the initial experiment and additionally in a single sample in experiment 3. For all the experiments presented here, the number of samples analysed is low. Nevertheless, because we observe positive evidence of pollen DNA carryover after the various treatments, even with small sample numbers, the results are still informative observations. Designing a statistically robust assessment of these effects would be a worthwhile future endeavour.

Dietary analysis of plant-feeding insects relies on successfully eliminating surface contamination. If this cannot be performed reliably, then it will not be possible to differentiate between plants that the insect is feeding on and plants the insect has been in contact with. Pollen is likely to be of particular concern, as it has a robust outer coating that resists simple decontamination processes, and some pollen types (notably pollen from insect-pollenated plants) are sticky and will adhere to insects. Moreover, pollen is known to travel large distances [31], so contamination is not necessarily reflective of an insect’s local environment. In the absence of adequate controls, these contamination events can seem to be biologically plausible dietary results. 

Our results assume that the majority of surface contamination observed originates from pollen adhering to the surface of *P. spumarius*. Based on the results of our initial experiment (experiment 1), we designed a series of further experiments (experiments 2, 3, and 4) to try and develop a method to remove pollen from insects or render pollen DNA undetectable by qPCR. Due to the seasonal availability of different pollen types and our desire to coat insects with pollen from species they were highly unlikely to have encountered, we used a variety of different pollen types in our experiments. We were able to confirm that pollen will persist on the surface despite following both procedures outlined in Cooper et al. [5] and with the use of products such as Distel (experiment 3). The results from experiment 3 show that no plant material was identified from any replicate of the gut-dissected control group, suggesting that any plant material ingested by *P. spumarius* remains below the limit of detection for this method. As *P. spumarius* is a xylem feeder, we hypothesise that only a small amount of plant material is ingested upon the initial piercing of the plant cells, which is quickly degraded in the stomach of the insect. Indeed, the low levels of DNA in the stomach contents were confirmed in another *P. spumarius*-based study [29]. The lack of plant DNA is supported by the results of experiment 2—a more rigorous sterilisation procedure using Tween and bleach results in no detectable plant DNA. This result could be a ‘true’ result—no plant DNA was recovered from the gut as it was absent—or it could be the complete destruction or fragmentation of recoverable plant DNA. We note that the use of Distel did reduce the levels of pollen material identified from 91–99% in the controls to 40–55% (in experiment 3).

Experiment 3 highlights the issue of laboratory contamination when dealing with such sensitive methods, especially when dealing with a sample type as adhesive as pollen. Two sample types that were not dusted with tulip pollen were found to contain tulip pollen sequences. However, the tulip-dusted samples could still be differentiated from the non-dusted samples due to the lower levels of tulip present (both in the absolute reads and as a proportion of the total abundance). More specifically, (A) the number of replicates positive for tulip pollen was three out of three for every pollen-dusted sample, but lower in the non-dusted samples (none for two sample types, one out of three for one sample type, two out of three for one sample type); (B) the number of tulip reads was lower in the non-dusted samples than in the dusted samples; the contaminated, non-dusted samples had an average of 6711 tulip reads, the pollen-dusted samples had an average of 154,440 tulip reads, and the Distel-treated, pollen-dusted samples had an average of 48,860 tulip reads; and (C) the proportion of tulip reads is lower in the non-dusted samples than in the dusted samples: the sequences of the contaminated, non-dusted samples were between 2 and 9% tulip; the sequences of the dusted samples were between 92 and 100% tulip; and the sequences of the Distel-treated dusted samples were between 41 and 55% tulip (see Supplementary Materials S1, E3—Matrix). We therefore believe that the tulip sequences present on the tulip-dusted sample types are reflective of an inability to remove the tulip pollen rather than being laboratory contaminants. As such, these results are still informative.

Existing studies state that their proposed sterilisation techniques produce satisfactory results. However, some of these studies have been tested on dead plant material (chopped roots) [7] or non-plant material (blood) [8], which do not possess the adhesive properties or robustness of pollen. In some instances, bleach washes would most likely remove enough surface contamination to yield positive results but did not monitor for all environmental contaminants. Previous studies have investigated the detectability of pollen DNA in arthropod washing liquids [32], but the work presented here has the advantage of using artificially dosed pollen and, therefore, has the ability to detect pollen retained on an insect’s surface after washing. The pollen-dusting approach used may introduce pollen at higher levels than would be expected in nature, so future work could include an assessment of sterilisation approaches at lower levels of pollen contamination. However, given a lack of a priori knowledge of the external pollen contamination of any wild-caught insect that would eventually be the target of a gut content analysis, it is preferable to have a method that can remove external pollen at the highest levels of contamination.

In summary, despite the plausible results from the initial experiment (detection of DNA from the plant species that the insects were feeding on), we found we could not remove surface contamination from the insect while retaining signal from the guts. Our results demonstrate the great difficulty of removing external contamination, particularly involving pollen, and in these instances, the efficacy of surface sterilisation methods should be demonstrated. For studies where the presence of contamination will have an impact on the output, great care should be taken when interpreting the results of studies that do not use surface sterilisation (e.g., [29]) or that use the methods that we have assessed here.

## Figures and Tables

**Table 1:** Primers used for the amplification of the *rbcLa* amplicon [12,13], used in experiment 1. Primers and probes used for COX [14,15] and NAD [16,17] real-time PCR assays, used in experiment 2. Primers used for the amplification of the *rbcLa* [12,18] amplicon, used in experiment 3. Min tag and Nextera tag sequences are in lowercase. These allow the index tags to be added to the samples during library preparation to allow for the discrimination of individual samples following sequencing.

Primer	Sequence (5′–3′)
Min-rbcLa-F ^a^	tttctgttggtgctgatattgcTGTCACCACAAACAGAGACTAAAGC
Min-rbcLa-R ^a^	acttgcctgtcgctctatcttcGTAAAATCAAGTCCACCRCG
COX-F ^b^	CGTCGCATTCCAGATTATCCA
COX-R ^b^	CAACTACGGATATATAAGRRCCRRAACTG
COX-P ^b^	AGGGCATTCCATCCAGCGTAAGCA
*Nad5*-F ^b^	GATGCTTCTTGGGGCTTCTTGTT
*Nad5*-R ^b^	CTCCAGTCACCAACATTGGCATAA
*Nad5*-P ^b^	AGGATCCGCATAGCCCTCGATTTATGTG
Nex-rbcLa-F ^c^	tcgtcggcagcgtcagatgtgtataagagacagATGTCACCACAAACAGAGACTAAAGC
Nex-rbcLa-R ^c^	gtctcgtgggctcggagatgtgtataagagacagCGGTCCAYACAGYBGTCCAKGTACC

^a^ Experiment 1. ^b^ Experiment 2. ^c^ Experiment 3.

**Table 2:** Treatments applied to *P. spumarius* in experiment 3.

Sample	Sample Type	Replicates	Pollen	Sterilisation	Purpose
1	Whole pollen-dusted *P. spumarius*—intact	3	Tulip	None	Controls
2	Whole pollen-dusted *P. spumarius*—intact	3	Tulip	Distel	Distel sterilisation
3	Guts dissected from pollen-dusted *P. spumarius*	3	Tulip	None	To check contamination from surface in dissected guts
4	Associated exoskeletons from dissected pollen-dusted *P. spumarius* ^a^	3	Tulip	None	Controls
5	Whole non-pollen-dusted *P. spumarius*—intact	3	None	None	Controls
6	Whole non-pollen-dusted *P. spumarius*—intact	3	None	Distel	Distel sterilisation controls
7	Guts dissected from non-pollen-dusted *P. spumarius*	3	None	None	Detection of natural food from the environment
8	Associated exoskeletons from dissected non-pollen-dusted *P. spumarius* ^b^	3	None	None	Detection of natural food contamination of surface of insect
9	Pollen only, in tubes	3	Tulip	None	Controls—ability to extract pollen DNA and thus detect it in treatments

^a^ Exoskeletons were taken from sample 3. ^b^ Exoskeletons were taken from sample 7.

**Table 3:** Cycling threshold (CT) values for the COX and NAD TaqMan assays, targeting the gut contents and pollen, respectively. Higher values indicate lower levels of DNA; the maximum number of cycles was 40, so values of 40 can be considered ‘no detection’).

Treatment	Pollen	Number of Repeats	Average COX CT	Average NAD CT
Tween/Bleach	Coated	1	37.11	40
		2	40	40
	Uncoated	1	38.78	40
		2	40	40
Tween	Coated	1	36.55	39.47
	Uncoated	1	34.55	40
Untreated	Coated	1	33.12	36.69
	Uncoated	1	32.07	40
Extraction blank	-	-	40	40
COX-positive control	-	-	25.53	-
NAD-positive control	-	-	-	27.30
Negative control	-	-	40	40

**Table 4:** Samples detailing where tulip pollen was found to be present or absent.

Sample	Sample Type	Pollen	Sterilisation	Replicates Tulip Detected in
1	Whole pollen-dusted *P. spumarius*—intact	Tulip	None	3/3
2	Whole pollen-dusted *P. spumarius*—intact	Tulip	Distel	3/3
3	Guts dissected from pollen-dusted *P. spumarius*	Tulip	None	3/3
4	Associated exoskeletons from dissected pollen-dusted *P. spumarius* ^a^	Tulip	None	3/3
5	Whole non-pollen-dusted *P. spumarius*—intact	None	None	0/3
6	Whole non-pollen-dusted *P. spumarius*—intact	None	Distel	1/3
7	Guts dissected from non-pollen-dusted *P. spumarius*	None	None	0/3
8	Associated exoskeletons from dissected non-pollen-dusted *P. spumarius* ^b^	None	None	2/3
9	Pollen only, in tubes	Tulip	None	3/3

^a^ Exoskeletons were taken from sample 3. ^b^ Exoskeletons were taken from sample 7.

**Table 5:** CT values for NAD TaqMan assay, targeting pollen DNA. Higher values indicate lower levels of DNA.

Time under UV (Minutes)	NAD—CT1	NAD—CT2	NAD—Average CT
0	26.73	26.41	26.57
15	29.81	30.18	30
30	30.40	30.38	30.39
60	33.36	33.03	33.20
120	34.69	34.51	34.60
240	36.61	37	36.81
Extraction Blank	40	40	40
Positive Control	25.95	26.11	26.03
Negative Control	40	40	40

## Data Availability

Data have been made available on the Sequence Read Archive (SRA) under BioProjectID PRJNA1066743.

## Supplementary Materials

The following are available online at: https://www.mdpi.com/article/10.3390/insects15100732/s1, S1—Abundance Matrices.
